# Microgripper Robot with End Electropermanent Magnet Collaborative Actuation

**DOI:** 10.3390/mi15060798

**Published:** 2024-06-17

**Authors:** Yiqun Zhao, Dingwen Tong, Yutan Chen, Qinkai Chen, Zhengnan Wu, Xinmiao Xu, Xinjian Fan, Hui Xie, Zhan Yang

**Affiliations:** 1 School of Mechanical and Electrical Engineering, Soochow University, Suzhou 215131, China; 2129401067@stu.suda.edu.cn (Y.Z.); 2129401038@stu.suda.edu.cn (D.T.); chenqinkai0426@163.com (Q.C.); 20225229110@stu.suda.edu.cn (Z.W.); yangzhan@suda.edu.cn (Z.Y.); 2 School of Future Science and Engineering, Soochow University, Suzhou 215222, China; 2262405036@stu.suda.edu.cn (Y.C.);; 3 State Key Laboratory of Robotics and Systems, Harbin Institute of Technology, Harbin 150080, China

**Keywords:** actuation system, soft robot, microgripper, magnetic actuation

## Abstract

Magnetic microgrippers, with their miniaturized size, flexible movement, untethered actuation, and programmable deformation, can perform tasks such as cell manipulation, targeted drug delivery, biopsy, and minimally invasive surgery in hard-to-reach regions. However, common external magnetic-field-driving devices suffer from low efficiency and utilization due to the significant size disparity with magnetic microgrippers. Here, we introduce a microgripper robot (MGR) driven by end electromagnetic and permanent magnet collaboration. The magnetic field generated by the microcoils can be amplified by the permanent magnets and the direction can be controlled by changing the current, allowing for precise control over the opening and closing of the magnetic microgripper and enhancing its operational range. Experimental results demonstrate that the MGR can be flexibly controlled in complex constrained environments and is highly adaptable for manipulating objects. Furthermore, the MGR can achieve planar and antigravity object grasping and transportation within complex simulated human cavity pathways. The MGR’s grasping capabilities can also be extended to specialized tasks, such as circuit connection in confined spaces. The MGR combines the required safety and controllability for in vivo operations, making it suitable for potential clinical applications such as tumor or abnormal tissue sampling and surgical assistance.

## 1. Introduction

In recent years, microrobots have developed a wide variety of actuators [1,2,3,4,5], making them more and more widely used in biomedical and environmental applications (BME) [6,7,8,9,10,11,12]. The microgripper, as a representative microrobot, can easily grasp, release, and manipulate tiny objects thanks to its bionic design mimicking the human hand structure [13], which makes it a popular research direction in the field of micro–nanomanipulation [14,15]. Microgrippers can be classified into tethered and untethered through their actuation mechanism for shape transformation. The main driving methods of tethered microgrippers are piezoelectric [16], thermoelectric [17], electrostatic [18], electromagnetic [19], and shape memory alloy (SMA) [20]. The main driving methods of untethered microgrippers are thermally responsive microgrippers [21,22], magnetically driven microgrippers [23], chemically responsive microgrippers [24], optically driven microgrippers [25], etc. Among these actuation methods, magnetic actuation is considered the most promising due to its great permeability, being harmless to tissues, being remote and wireless, high control precision, and multiple manipulation modes to offer multiple degrees of freedom of locomotion [13]. Therefore, a large number of magnetic microgrippers have been developed successively. Xueqi Li et al. used cellulose nanofibers through a papermaking process, added sodium alginate and BaFe_12_O_19_ particles to synthesize M-films with good foldability, origami, and magnetism, and constructed a multimodal magnetic gripper robot with the functions of programmable movement, grasping and transporting goods, and wireless operation through origami magnetization [26]. Gangsheng Chen et al. constructed a material with multiple programmable temperatures, as the composition of the low-melting alloy (LMA) changes by encapsulating magnetic NdFeB particles with the LMA and embedding them in an elastomer, and the material can be used to fabricate bio-inspired tracks with multimodal motions, reconfigurable robotic grippers capable of adapting to grasping, and reconfigurable electronic circuits [27]. Zhaoxin Li et al. reported a novel multilayer for patterning magnetic nanoparticles in an ultraviolet (UV) cured polymer matrix 3D printing technique, which can be used to achieve shape changes in the robot, including grasping, rolling, swimming, and walking, induced by the global actuation field by programming heterogeneous magnetization within discrete multilayer robotic segments [28]. Yuanxi Zhang reported a coaxial printing method to prepare a hybrid magnetic–mechanical–electrical (MME) core–sheath fiber, which can be realized by including programmable magnetization, somatosensory, and magnetic actuation, along with simultaneous wireless energy transfer [29]. However, most of the common magnetic microgrippers are currently controlled by applying a magnetic wrench through an external magnetic field source, and to generate sufficient magnetic fields in deep regions, such as the heart or the stomach, it needs to be actuated using an electromagnet that is orders of magnitude larger than the workspace [30]. This undoubtedly places high demands on both the experimental platform and the practical application scenarios.

Electromagnetic actuators actuate magnetized objects through the interaction of electric and magnetic fields. Due to its high efficiency and performance, a large amount of research on electromagnetic actuators has appeared [31,32,33,34,35]. However, the existence of scaling laws makes the performance of electromagnetic actuators on small scales not ideal [36]. Nevertheless, micro-electro-magnetic actuators still have the characteristics of low driving voltage [37], high output current [38], long lifetime [39], and low complexity [40,41], which makes micro-electro-magnetic actuators widely used in minimally invasive surgery, drug delivery, and microfluidic systems. Jakub Sikorski et al. built a Microrobotic Infrastructure Loaded into a Magnetically Actuated Catheter (MILiMAC) using three miniaturized electromagnets to achieve the creation of a two-dimensional target workspace to actuate a wide range of microagents within the body [30]. Yue Wang et al. achieved a high-force-density micro-electro-magnetic actuator with a long current time input by designing a mesh microcoil with a multiturn structure [42]. Thanh Nho Do et al. reported a miniature soft EMF actuator made of a silicone polymer, a liquid metal (LM) alloy (eutectic gallium indium, EGaIn), and a magnetic (NdFeB) powder, whose ability to accommodate high currents and forces has allowed for applications in vibro-haptic feedback displays and miniature soft robotic grippers [43]. It can be seen that micro-electro-magnetic actuators can still occupy a place in the field of microactuators with their own characteristics. The micro-electro-magnetic actuator replaces the external large electromagnet for the shape transformation drive control of the microgripper, which can significantly improve the utilization of the workspace, reduce the occupancy rate of the external device volume, reduce the cost of the drive control, and improve the accuracy of the control.

**Figure 1:**
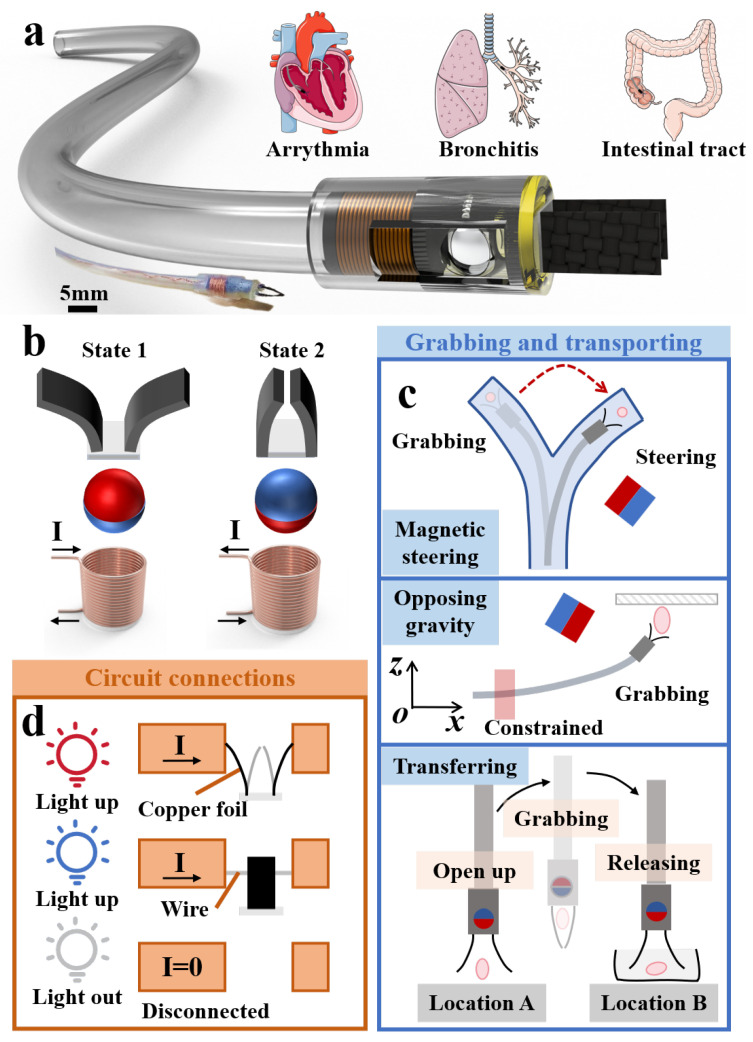
Application scenarios and capabilities of the MGR: (**a**) real-life photograph and structural rendering of the MGR, along with its application scenarios; (**b**) basic gripping mechanism of the MGR; (**c**) MGR’s capability to grip and transport objects; (**d**) MGR’s ability to connect circuits in constrained environments.

In this article, we report a microgripper robot (MGR) driven by distal electropermanent magnet collaboration, as illustrated in Figure 1a. Since the opening and closing actions of common magnetic microgrippers are currently controlled by an external magnetic field, their working space is restricted by the external control platform, especially when driven by an enclosed magnetic control platform. However, our end device, as shown in Figure 1b, utilizes a permanent magnet sphere that rotates in the direction of the microcoil’s magnetic field to amplify the magnetic field strength. This controls the opening and closing of the magnetic gripper, removing the working space limitations of the external control platform and allowing the magnetic gripper to grasp target objects at any position. Additionally, the presence of the end permanent magnetic ball enables the MGR to perform locomotion actuation in response to an external magnetic field. As shown in Figure 1c, the MGR is capable of grasping and transporting objects and can perform gripping tasks against gravity in space. We have verified that the MGR can grasp objects weighing ten times more than the microgripper itself, making it suitable for applications such as retrieving ingested foreign objects and sampling pathological tissues in the gastrointestinal tract. Moreover, we conducted safety temperature evaluations and maneuverability assessments under complex constraints, ensuring its potential value for applications within the human body. Furthermore, we constructed a planar circuit model to examine the MGR’s manipulability and scalability under constraints, as shown in Figure 1d. Finally, we developed two-dimensional and three-dimensional complex trajectory gripping models to demonstrate the MGR’s capability to grasp objects within simulated human cavities.

## 2. Materials and Methods

### 2.1. Design of MGR

**Figure 2:**
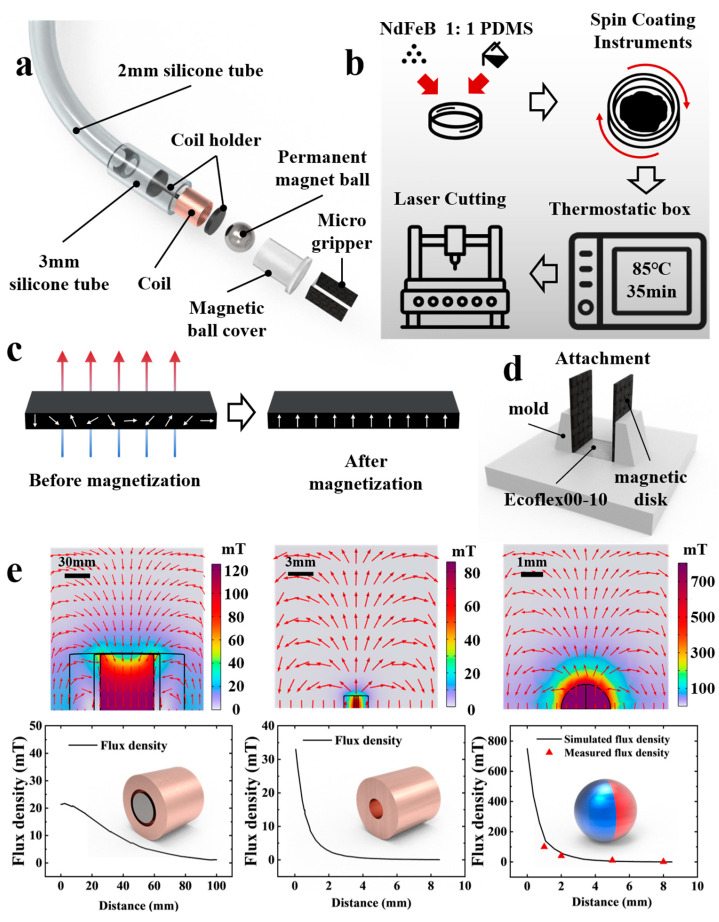
Structure and fabrication process of the MGR: (**a**) Exploded view of the MGR’s structural components; (**b**) fabrication process of the magnetic sheets in the MGR; (**c**) principle of magnetization of magnetic sheets; (**d**) method of connecting the magnetic sheets in the MGR; (**e**) comparative analysis of magnetic fields of core amplified external electromagnetic coils, internal microcoils, and permanent magnetic balls.

The structure of the MGR, as shown in Figure 2a, primarily consists of an end magnetic microgripper and a micro-electro-magnetic driving system. The end magnetic microgripper can perform two actions, opening and closing, driven by an external magnetic field. To expand the working scenarios of the magnetic microgripper, it needs to achieve a wider operating angle to grasp larger objects. Therefore, we abandoned the commonly used integrated magnetic microgripper design and developed a hybrid material-connected magnetic microgripper. This magnetic microgripper is mainly composed of two oppositely magnetized magnetic sheets connected by silicone, forming a three-dimensional actuation mechanism. Compared with the integrated design, it can achieve a larger range of opening and closing angles and significantly greater end-grasping force under the same external field.

The micro-electro-magnetic driving system comprises a microcoil and a permanent magnet ball controlled by the microcoil. The microcoil is connected to an external power source through a catheter, generating magnetic fields in different directions by altering the current direction. Due to the magnetic torque, the permanent magnet ball rotates, and its magnetic moment direction remains aligned with the center of the magnetic field, amplifying the magnetic field intensity generated by the microcoil. Magnetic field simulations (COMSOL Multiphysics 6.0, COMSOL, Stockholms, Sweden) were carried out on the magnetic fields of the core amplified external coil, the internal microcoil, and the permanent magnetic sphere, and the results are shown in Figure 2e. Comparative analysis reveals that the magnetic field of the permanent magnetic sphere is significantly stronger than that of the conventional external electromagnetic coil driver and the internal microcoil, which enables the magnetic microgripper to be subjected to a sufficiently large magnetic torque to complete the gripping action in the working scenario. Since the magnetic field produced by the microcoil undergoes distance attenuation, its magnitude at the same location is not comparable with the magnetic field of the permanent magnetic sphere. Therefore, it does not affect the ability of the permanent magnetic sphere to control the magnetic microgripper. Additionally, the permanent magnet ball can guide the MGR to the target working scenario under the influence of an external gradient magnetic field, enhancing the precision of MGR control.

With the combination of the end magnetic microgripper and the micro-electro-magnetic driving system, the MGR can complete active target operations in complex and constrained spaces through a method of combined internal and external control.

### 2.2. Preparation of MGR

The core components of the micro-electro-magnetic driving system are the coil and the permanent magnet ball. To align the rotation direction of the magnet ball with the magnetic field direction generated by the coil, we need to maximize the number of coil turns, which also increases the coil’s cross-sectional area. However, an excessively large coil cross-section would increase the robot’s outer diameter, restricting its operational scenarios. Considering these factors, we set the coil parameters as follows: inner diameter of 0.5 mm, outer diameter of 3 mm, 100 turns, and a single wire diameter of 0.16 mm. To amplify the coil’s magnetic field as much as possible within the limited space, we selected a permanent magnet ball with a diameter of 3 mm.

Given that the supporting part of the coil frame is only a 0.5 mm diameter cylinder, we used a high-hardness photosensitive resin (Tough 2000 Resin, Formlabs, Somerville, MA, USA) to 3D print the coil frame. We then printed a transparent resin (Water-Wash Resin+, Anycubic, Shenzhen, China) cover for the permanent magnet ball, allowing for external observation and ensuring that the ball only contacts its inner wall during rotation, minimizing friction and ensuring the alignment of the ball’s magnetic field with the coil-generated magnetic field. 

It is important to note that the magnetic field of the permanent magnet ball diminishes significantly with distance. Thus, the wall thickness of the magnet ball cover should be printed as thin as possible to reduce the separation distance between the permanent magnet ball and the magnetic microgripper, thereby minimizing magnetic field attenuation. While the magnetic microgripper is in close contact with the permanent magnetic sphere, based on the hysteresis loop of the main magnetic material of the magnetic microgripper, neodymium iron boron, it can be concluded that the magnetic microgripper requires at least a 1 T magnetic field to change its magnetization direction [44]. Therefore, it will not be affected by the permanent magnetic sphere’s magnetization. Finally, we enclosed the entire actuation device in a transparent silicone tube with an inner diameter of 3 mm and used a 2 mm inner diameter silicone tube for the MGR’s body, connecting the actuation device to the external working scenario. The two silicone tubes with different inner diameters were connected and installed using silicone adhesive (KN-300, KANGLIBANG, Shenzhen, China). This variable cross-section design reduces the MGR’s bending stiffness, increasing its maneuverability in complex environments and expanding the applicable operational scenarios.

The overall preparation process for the magnetic microgripper is illustrated in Figure 2b. First, we evenly mix NdFeB particles (MQFP-15-7, Magnequench, Toronto, ON, Canada), polydimethylsiloxane (PDMS) prepolymer, and PDMS curing agent (Sylgard 184, Farnell, Leeds, UK) in a ratio of 6:5:1 and pour the mixture into a petri dish. Using a spin coater (KW-4A, SETCAS, Beijing, China) at various spin speeds and times, we obtain mixtures of different thicknesses, followed by a 5 min vacuum degassing process with a vacuum pump. The mixture is then placed in an oven at 85 °C for 35 min for curing. After curing, we use a laser cutter (D1 Pro, xTool, Irwindale, CA, USA) to shape the magnetic sheets into the specified forms. To achieve the desired magnetization direction for the processed magnetic sheets, we 3D print auxiliary magnetization molds corresponding to the magnetization direction. The sheets are then placed in a capacitive pulse power source (J1801N, Golden Wolf, Nanjing, China) and magnetized under a pulsed magnetic field of approximately 1.8 T. It is important to note that we conducted magnetization experiments in different directions and found that when the magnetization direction is perpendicular to the surface of the magnetic sheet, as shown in Figure 2c, the magnetic sheets driven by the permanent magnet ball achieve the maximum opening and closing angles within the operating range.

To assemble the magnetized sheets, we used photosensitive resin to 3D print a connecting mold, as shown in Figure 2d. The mold is precoated with silicone release agent (Ease Release^®^ 200, Smooth-On, Macungie, PA, USA), and the two magnetized sheets are placed on opposite sides of the mold with their magnetization directions reversed. To enhance the magnetic microgripper’s response to the magnetic field, the connecting medium should be made from a material with a low Young’s modulus. Therefore, we inject a 1:1 volume ratio mixture of silicone rubber (Ecoflex™ 00-10, Smooth-On, Macungie, PA, USA) into the central recess using a syringe and then place the mold in an oven at 70 °C for 40 min for curing, thus completing the connection of the magnetic sheets. After preparing the magnetic microgripper, we use silicone adhesive to bond the microgripper to the permanent magnet ball cover, thereby completing the fabrication process of the entire robot.

### 2.3. Modeling of MGR’s Bending Characteristics

The MGR’s locomotion actuation is primarily caused by the response of the terminal permanent magnet ball to the magnetic field, which induces bending in the body part’s rod structure. Therefore, we modeled the MGR’s steering motion under the influence of the magnetic field based on fundamental magnetic principles and the Euler–Bernoulli beam assumption.

#### 2.3.1. Magnetic Field Response of Permanent Magnetic Balls

**Figure 3:**
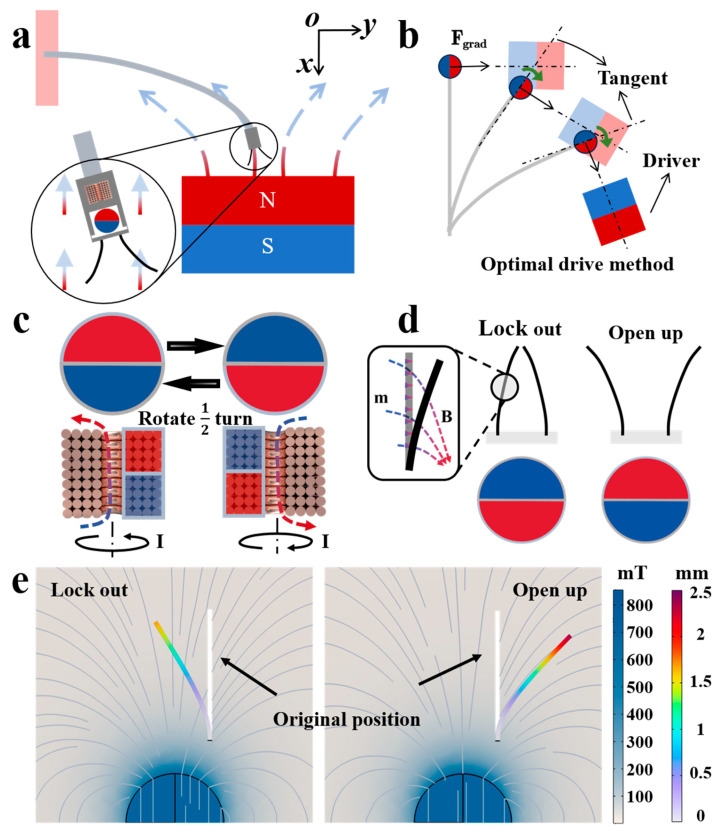
Theoretical analysis and simulation results of MGR bending and magnetic microgripper opening and closing: (**a**) schematic diagram of the MGR deflection principle under an external magnetic field; (**b**) optimal driving method for the MGR; (**c**) method of controlling the direction of the permanent magnet ball using a microcoil; (**d**) working principle of the permanent magnet ball controlling the opening and closing of the magnetic microgripper; (**e**) simulation results of the deformation of the magnetic microgripper under the influence of the permanent magnet ball. The first color scale represents the displacement analysis of the magnetic field distribution, and the second color scale represents the displacement magnitude of the magnetic microgripper relative to its initial position.

Compared with electromagnets, permanent magnets can generate strong magnetic fields. Considering that the strength and distribution of the magnetic field depend on the size and shape of the magnet [45], we chose to use a cylindrical permanent magnet with a diameter of 50 mm and a height of 30 mm to control the deflection of the MGR. To facilitate the calculation of the magnetic field’s magnitude and direction at a specific point, we approximate the permanent magnet as a point dipole source located at the center of the magnet’s volume. The magnetic field $B\left(p\right)$ generated by the dipole at position p is given by

(1) $$B(p)=\frac{\mu_{0}}{4\pi ||r|{|}^{3}}(\frac{3rr^{T}}{||r|{|}^{2}}-I)M$$

where $\mu_{0}$ is the vacuum permeability, r is the vector pointing from the center of the magnet to the controlled device, I is the unit matrix, and M is the magnetic moment of the dipole source. In the presence of this magnetic field, the magnetic force and moment applied to the end permanent magnet sphere are described below:

(2) $$\overset{\to }{F}=(\overset{\to }{m}\cdot \nabla )\overset{\to }{B}$$

(3) $$\overset{\to }{T}=\overset{\to }{m}\times \overset{\to }{B}$$

where B is the magnetic induction strength to which the dipole is subjected and m is the magnetic moment of the dipole. When the external magnetic field is nonuniform, the magnetic field has a magnetic gradient, and the magnetic ball is subjected to both magnetic moment and magnetic force [46]. In this case, since the permanent magnetic ball is placed in a permanent magnetic ball cover causing it to rotate freely, and its rotation due to the magnetic moment does not deflect the MGR. However, the free-rotating characterization of the permanent magnet ball causes that in the established dipole model, the dipole magnetic moment should always be aligned with the direction of the external magnetic field, which is the x-direction, as shown in Figure 3a. Therefore, we can express the magnetic moment as

(4) $$\overset{\to }{m}=\left[\begin{array}{c} 1\\ 0\\ 0 \end{array}\right]m=m_{x}$$

We further decompose and simplify Equation (2) to obtain the gradient force on the permanent magnetic sphere in the external magnetic field as

(5) $$\vec{F}=\left[\begin{array}{ccc} \frac{\partial B_{x}}{\partial x} & \frac{\partial B_{y}}{\partial x} & \frac{\partial B_{z}}{\partial x}\\ \frac{\partial B_{x}}{\partial y} & \frac{\partial B_{y}}{\partial y} & \frac{\partial B_{z}}{\partial y}\\ \frac{\partial B_{x}}{\partial z} & \frac{\partial B_{y}}{\partial z} & \frac{\partial B_{z}}{\partial z} \end{array}\right]\left[\begin{array}{c} m_{x}\\ m_{y}\\ m_{z} \end{array}\right]=\left[\begin{array}{c} \frac{\partial B_{x}}{\partial x}\\ \frac{\partial B_{x}}{\partial y}\\ \frac{\partial B_{x}}{\partial z} \end{array}\right]m_{x}$$

#### 2.3.2. Bending Characteristics of MGR

According to the Euler–Bernoulli beam assumption, the conduit can be modeled as a beam model with uniform bending stiffness [47]. Assuming that the beam is inextensible over the range of forces studied here, we define the arc length of the MGR as s in Lagrangian coordinates (s=0 at the front end and s=l at the tip) to obtain the following equation:

(6) $$EI\frac{\partial^{2}\theta }{\partial s^{2}}=-Fcos\theta$$

where E represents Young’s modulus, I represents area moment of inertia, and θ represents the angle before and after beam deformation. And the boundary conditions are

(7) $$\theta {|}_{s=0}=0,EI\frac{\partial \theta }{\partial S}{|}_{s=l}=0$$

Since the magnetic moment of the permanent magnet ball aligns with the external magnetic field, the direction of the gradient force experienced by the MGR varies with the deflection angle and is not always perpendicular to the end. Therefore, as shown in Figure 3b, when the magnetic field direction is perpendicular to the MGR’s end, the tangential force exerted on the MGR due to the gradient force is maximized, resulting in the greatest degree of bending and representing the optimal driving state.

### 2.4. Modeling of MG’s Motion Behavior

As previously mentioned, controlling the magnetic gripper’s opening and closing using an external magnetic field results in low workspace utilization and energy efficiency. Therefore, to enable direct control of the magnetic gripper at the MGR’s end, we adopted the following control strategy.

The microcoil is connected to an external power source through the body of the MGR, generating a magnetic field when electrified. To investigate the magnetic properties of the microcoil, we established an electromagnetic model based on the Biot–Savart law to study the influencing factors of the electromagnetic field and force. When current flows through the microcoil mounted on the MGR, the magnetic field $B\left(r\right)$ at a specific point can be calculated using the following formula:

(8) $$B(r)=\frac{\mu_{0}}{4\pi }\int_{C}\;\frac{Idl\times r}{|r{|}^{3}}$$

where $B\left(r\right)$ is the magnetic field, I is the current, l is the length of the coil, and $\mu_{0}$ is the magnetic constant. As shown in Figure 3c, changing the direction of the current in the microcoil alters the direction of the generated magnetic field. The permanent magnet ball at the MGR’s end responds to the microcoil’s magnetic field and rotates by half a turn under the influence of magnetic torque, aligning its magnetic moment with the direction of the microcoil’s magnetic field. Under the amplified magnetic field from the permanent magnet ball, as illustrated in Figure 3d, the magnetic gripper experiences magnetic torque, causing it to either close or open, thus performing the grasping action.

The bending properties of the magnetic gripper primarily depend on the PDMS polymer used in constructing the magnetic sheets. The stress–strain relationship of PDMS exhibits high nonlinearity, which should be described using a hyperelastic model. Therefore, we established the following equation based on the neo-Hookean model:

(9) $$W=\frac{\mu }{2}(I_{1}-3)+\frac{1}{D_{1}}(J-1{)}^{2}$$

(10) $$\sigma =\mu ((1+\varepsilon_{1})-\frac{1}{(1+\varepsilon_{1}{)}^{2}})$$

where W represents the elastic strain energy potential, μ is the shear modulus, $I_{1}$ is the first invariant of the right Cauchy–Green deformation tensor, $D_{1}$ is a material constant, and $\varepsilon_{1}$ is the strain. To further validate the deformation and bending degree of the magnetic gripper, as shown in Figure 3e, we simulated the degree of bending that the magnetic microgripper can achieve when opening and closing on one side, with the magnetic moment of the terminal permanent magnet ball pointing in different directions. The simulation results indicate that the magnetic field of the permanent magnet ball is not uniform and is relatively divergent. Consequently, the magnetic torque experienced by the magnetic microgripper when opening is greater than when closing. This characteristic enhances the graspable range of the magnetic microgripper and expands its operational scenarios.

## 3. Results

### 3.1. Coil Safety Temperature Evaluation

**Figure 4:**
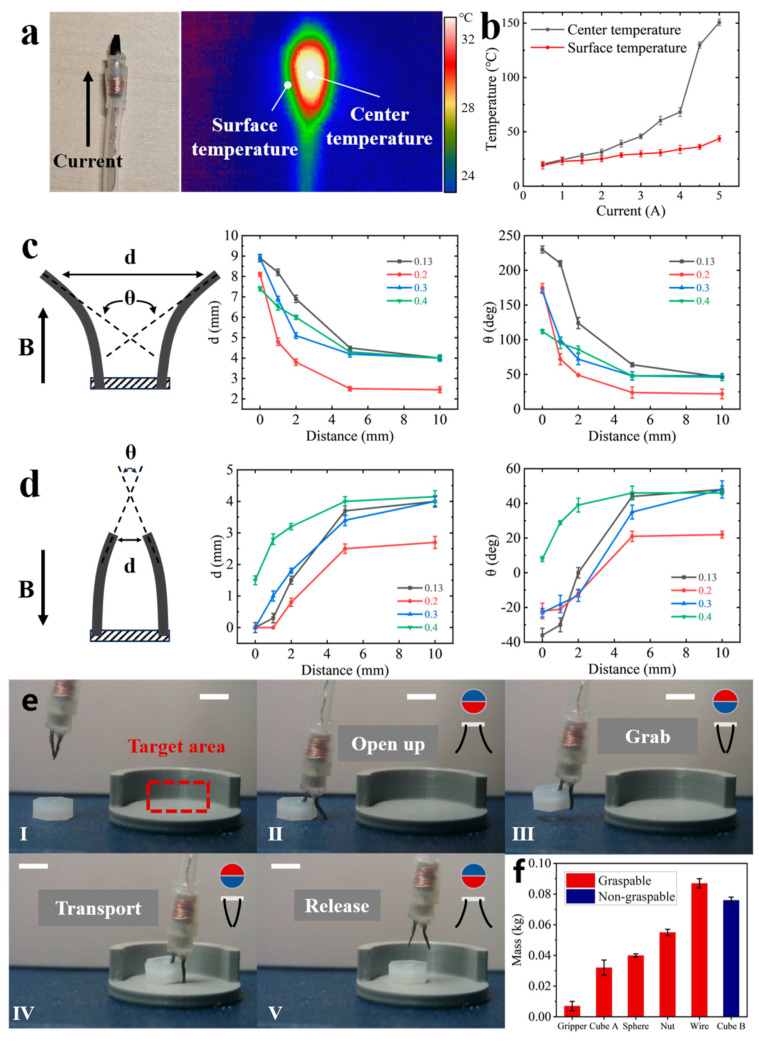
Safety temperature assessment, bending degree evaluation, and graspable object evaluation of the MGR: (**a**) real object and thermal imaging photos taken during the safety temperature assessment using a thermal imager; (**b**) center and surface temperatures of the MGR under different driving currents; (**c**) opening distance and angle of magnetic microgrippers of different thicknesses driven by a 3 mm permanent magnet ball; (**d**) closing distance and angle of magnetic microgrippers of different thicknesses driven by a 3 mm permanent magnet ball; (**e**) Grasping and transporting a plastic nut by the MGR: (I) The target area for this grasping task is a small platform created using 3D printing, (II) Open the gripper to encircle the plastic nut, (III) Close the gripper to grasp the plastic nut, (IV) Transport the plastic nut to the target area, (V) Open the gripper to release the plastic nut. (**f**) grasping performance of the MGR for objects of different weights and volumes. Scale bars, 5 mm.

When the MGR performs grasping actions, the microcoil is energized with direct current (DC) to generate a magnetic field that controls the permanent magnet ball, causing the magnetic microgripper to open and close. Due to the small current and voltage of DC power, it poses no threat to the human body, even when operating internally. However, the microcoil generates significant heat when current flows through it, especially with high currents or very fine wire diameters. To ensure the safety of the robot during intra-coil applications, we analyzed the heating and heat transfer processes of the coil. We further evaluated the heating temperature of the microcoil at different currents using a thermal imaging camera (HM-TPK20-3AQF/W, HIKMICRO, Hangzhou, China), as shown in Figure 4a.

The heat generated by the coil primarily arises from the passage of current through the conductor (such as a metal coil). This is due to the presence of resistance within the conductor, causing collisions between free electrons and metal ions, thus generating heat energy Q. According to Joule’s law, this can be calculated as

(11) $$Q=I^{2}Rt$$

where I represents the magnitude of the current passed through, and t denotes the duration of the current flow.

The heat generated by the coil is conducted through the silicone tube to the surface, and the radial heat transfer equation under steady-state conditions, derived based on the Fourier heat transfer equation for a cylindrical geometry, can be utilized to calculate this as follows:

(12) $$T\left(r\right)=T_{s}+\frac{q}{4k}\left(R^{2}-r^{2}\right)$$

where $T_{r}$ represents the temperature at different radial positions on the silicone tube, $T_{s}$ denotes the surface temperature, q is the internal heat source intensity, R and r are the outer and inner radii, respectively, and k is the thermal conductivity of the silicone.

During normal operation, the microcoil is powered for 0.5 s, providing the magnetic ball sufficient time to overcome friction and rotate to the desired direction. To enhance the reliability of the temperature evaluation results, we measured the temperature after powering the microcoil for 1 s. Considering the actual working scenario, the primary medium for temperature transfer from the MGR to the internal environment is the outermost silicone tube. Therefore, we measured both the central temperature of the microcoil and the surface temperature of the outer silicone tube.

Maintaining a room temperature of 19 °C, with a wire diameter of 0.16 mm and 90 turns, the final measurement results are shown in Figure 4b. It can be seen that the central temperature of the microcoil rises rapidly with increasing current, reaching 150.7 °C within 1 s when 5A current is applied. In contrast, the surface temperature of the outer silicone tube increases more gradually, rising only by 24 °C even with the 5A current. However, this still poses a thermal safety risk for internal applications in the human body. Therefore, after considering the required magnetic field strength and safety within the human body, we chose to perform the grasping action with a current of 2A. With 2A current applied for 1 s, the central temperature increases to only 31.4 °C, while the surface temperature rises to 25.1 °C, which is an increase of just 6.1 °C from room temperature. This temperature rise is negligible in terms of tissue damage in internal studies [48]. Thus, in this study, the current for performing grasping actions is set at 2A.

Furthermore, considering the potential need for repeated grasping and releasing operations when using the MGR in vivo, it is essential to ensure that repeated current application under normal operating conditions does not generate excessive heat within the body, thus avoiding further tissue damage. To this end, we conducted an experiment to evaluate the heat dissipation rate of the microcoil during repeated energization. Specifically, we applied current to the microcoil twice, with a 1 s cooling interval between the two applications, and measured the surface temperature of the MGR after the second application. The experimental results, shown in Supplementary Materials Figures S5, indicate that under continuous energization, the surface temperature of the MGR significantly increases due to insufficient heat dissipation, doubling compared with the temperature observed with a single current application, which poses a risk of tissue damage. However, as the cooling time increases, the temperature after the second application gradually decreases, and at a cooling time of 52 s, the temperature increase is the same as that of a single current application.

### 3.2. Assessment of the Degree of Deformation of Magnetic Microgripper

Based on the aforementioned simulations of the bending degree of the magnetic microgripper, we conducted further experimental tests to optimize its final form. We used a spin coater to obtain magnetic sheets of different thicknesses (0.13 mm, 0.2 mm, 0.3 mm, 0.4 mm) to assemble magnetic microgrippers of varying thicknesses. Additionally, we utilized permanent magnet balls of different diameters (2 mm, 3 mm, 4 mm) to drive the grasper in various positions. As shown in Figure 4c,d, we defined the opening and closing distance at the end of the magnetic microgripper as d, and the opening and closing angle between the two magnetic sheets as θ. These two parameters were used as indicators to measure the opening and closing performance of the magnetic microgripper, and we conducted two sets of experiments to evaluate its opening and closing actions (measurement of the degree of opening and closing of the magnetic microgripper can be found at Supplementary Materials Figure S1).

The evaluation results of the bending degree driven by the 3 mm diameter permanent magnet ball are shown in Figure 4c,d, with the data from the other groups provided in Supplementary Materials Figures S3 and S4. It can be observed that during the opening process, the magnetic microgrippers with thicknesses of 0.13 mm and 0.3 mm exhibit the largest opening amplitude when positioned close to the permanent magnet ball, with angles exceeding 180°. When farther from the ball, the 0.2 mm thick microgripper shows significant deviation, while the others remain consistent. During the closing process, all except the 0.4 mm thick microgripper can close completely. The 0.13 mm and 0.2 mm thick microgrippers demonstrate strong closing ability when close to the ball, but when farther away, the 0.2 mm thick microgripper shows noticeable deviation, while the others remain consistent. Therefore, considering the experimental results, the 0.13 mm thick magnetic microgripper shows a clear advantage in bending performance. Consequently, this study continues to use the 0.13 mm thick magnetic microgripper in further experiments.

### 3.3. Assessment of Graspable Objects

Since the magnetic microgripper can be driven without impeding the normal movement of the MGR’s body parts, it can be used for targeted picking and placing tasks. However, the magnetic microgripper exhibits different grasping characteristics when faced with various objects. Therefore, we conducted grasping experiments using different-sized cubes, spheres, plastic nuts, plastic screws, and wires, as shown in Figure 4e. The primary task was to pick up objects from a flat surface and place them into a target area printed using a 3D printer (X1 Carbon, Bambu Lab, Shenzhen, China).

Figure 4e(I–V) details the process of controlling the magnetic microgripper to grasp a plastic nut by applying currents in different directions (detailed grabbing procedures for other objects can be found in Supplementary Materials Figure S2). In Figure 4e(II), the magnetic field generated by the microcoil is directed downwards, with the north pole of the permanent magnet facing down, causing the magnetic microgripper to open. In Figure 4e(III), with the current direction reversed, the south pole of the permanent magnet faces down, causing the magnetic microgripper to close and grasp the plastic nut. Figure 4e(IV,V) shows the release of the plastic nut using the same principle, thereby completing the targeted picking and placing task.

The grasping performance of the magnetic microgripper with respect to other objects is depicted in Figure 4f. It can be observed that the magnetic microgripper is capable of grasping objects weighing up to 10 times its own weight, demonstrating excellent grasping capability. However, it is noteworthy that the magnetic microgripper exhibits significantly superior grasping ability for wires compared with other block-shaped objects such as plastic nuts and spheres, while its performance in grasping cubes is the poorest. We attribute this to the magnetic microgripper’s preference for grasping smaller or elongated objects when faced with objects of equal weight. Conversely, objects with lighter weight but larger volume, which are less conducive to the magnetic microgripper’s grip, may also be unable to be grasped.

### 3.4. The Steering and Navigation Capabilities of MGR

**Figure 5:**
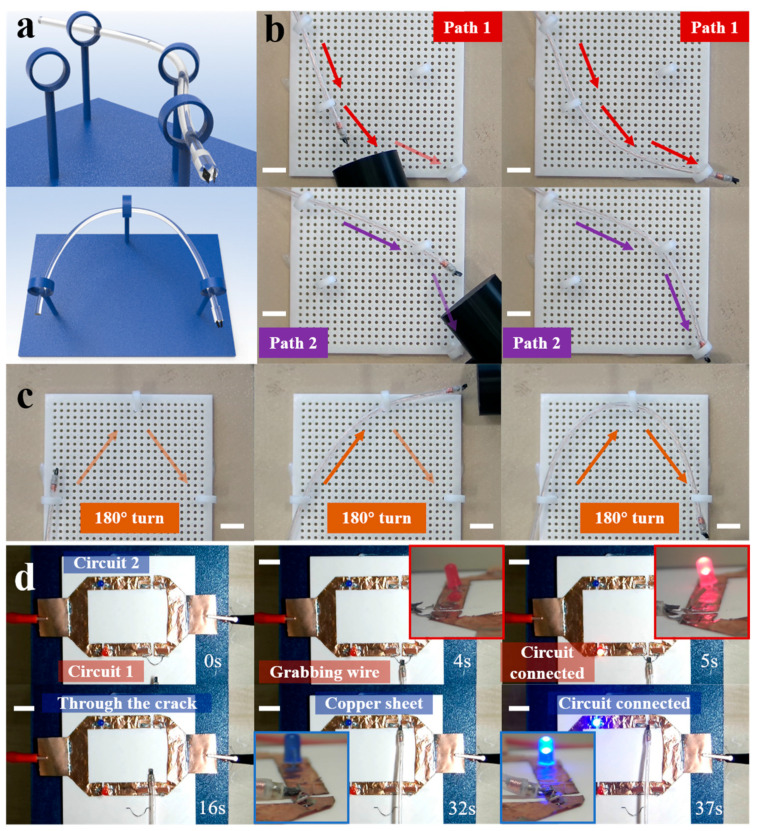
MGR’s steering experiment through rings and circuit connection experiment under external magnetic field guidance: (**a**) concept diagram of the MGR passing through rings and making a 180° turn; (**b**) MGR passing through two sets of rings via two different paths under external magnetic field guidance; (**c**) MGR completing a 180° turn experiment by passing through rings under external magnetic field guidance; (**d**) remote control of the MGR’s opening and closing to complete a circuit connection experiment in a confined space using two different working methods. Scale bars, 10 mm.

To assess the robot’s steering and navigation capabilities under the influence of external magnetic fields, we manipulate the permanent magnet to guide the deviation process. Figure 5a illustrates the concept of the MGR’s permanent magnet tip traversing a set of rings and making a 180° turn under the influence of an external magnetic field. Guided by the permanent magnet, the tip follows the gradient direction of the applied actuating field. As anticipated, the MGR traverses two selectable circular paths (indicated by red and purple arrows, respectively) to reach the same target position. As depicted in Figure 5b, we 3D-printed several 10 mm diameter rings and a corresponding base to construct an experimental setup for testing the MGR’s turning capabilities, simulating navigation through complex environments and constraints. We employed a cylindrical permanent magnet with a diameter of 50 mm and a height of 30 mm to apply the actuating magnetic field from a distance. The fundamental principle behind the MGR’s steering and turning is aligning the magnetic gradient direction with the desired direction to induce bending of the MGR’s tip towards the intended direction. Figure 5b and Movie S1 demonstrate steering achieved by manually manipulating a single permanent magnet, selectively navigating through two selectable circular paths. As the MGR’s body portion utilizes thinner 2 mm silicone tubing instead, it exhibits high flexibility and thinness, enabling it to achieve a 180° sharp turn on the same platform, as shown in Figure 5c. Both sets of experiments demonstrate the MGR’s outstanding navigation and steering capabilities.

### 3.5. Achieving Circuit Connection in Confined Environments

To explore the comprehensive control capabilities and extended application potential of the magnetic microgripper under the coordinated control of the permanent magnet sphere and microcoil at the end, we constructed a planar circuit experimental model. As depicted in Figure 5d and Movie S2, this experimental platform primarily consists of copper foil, with power supplied by 3.5 V, 0.5 A power sources at both ends. The circuit section comprises two parallel and disconnected branches, each with two LED lights soldered to indicate connectivity. The MGR employs different methods to establish connections for the two branches separately. Branch one requires the MGR to clamp the wires for connection, but a 5 mm wide gap is set in the middle of branch one, preventing the MGR from passing with the wires and thus constraining and limiting the MGR’s ability to establish circuit connections in the subsequent branch two. Therefore, branch two necessitates the MGR to alter its own conductivity properties, allowing circuit connectivity solely through gripping.

For the first working area of branch one, the MGR passes current to open the magnetic gripper, grasping the horizontally placed wire (3 s). Subsequently, the MGR continues to advance, causing the wire to make contact with the vertical copper foils on both sides, thereby closing the circuit. Meanwhile, the illumination of the red LED on the left indicates the normal closure of the branch (4 s). Upon completing the closure of branch one, the MGR releases the held wire on the left and maneuvers through the gap set up in branch one to reach the second working area of branch two (16 s).

Regarding the closure strategy for branch two, we opt to affix microcopper pieces onto the surface of the MGR’s magnetic gripper using silicone adhesive, enabling conductivity. As the wires near the copper foil joints, we first pass current to open the magnetic gripper, enclosing both ends of the wires (32 s). Then, reversing the current closes the magnetic gripper, and the circuit is completed through the copper pieces on the surface of the magnetic gripper. The illumination of the blue LED on the left confirms the normal closure of the branch (37 s).

This experiment demonstrates that MGR maintains good operational capability and executability in constrained environments. The magnetic gripper of the MGR exhibits adaptive grasping abilities across various specialized fields and for different materials and shapes of target objects. Additionally, by adjusting the material properties of the magnetic gripper, such as fabricating wettable structures on its surface or implementing biomimetic designs to increase friction, its application in diverse working scenarios can be further expanded.

### 3.6. Planar and Antigravity Grasping

**Figure 6:**
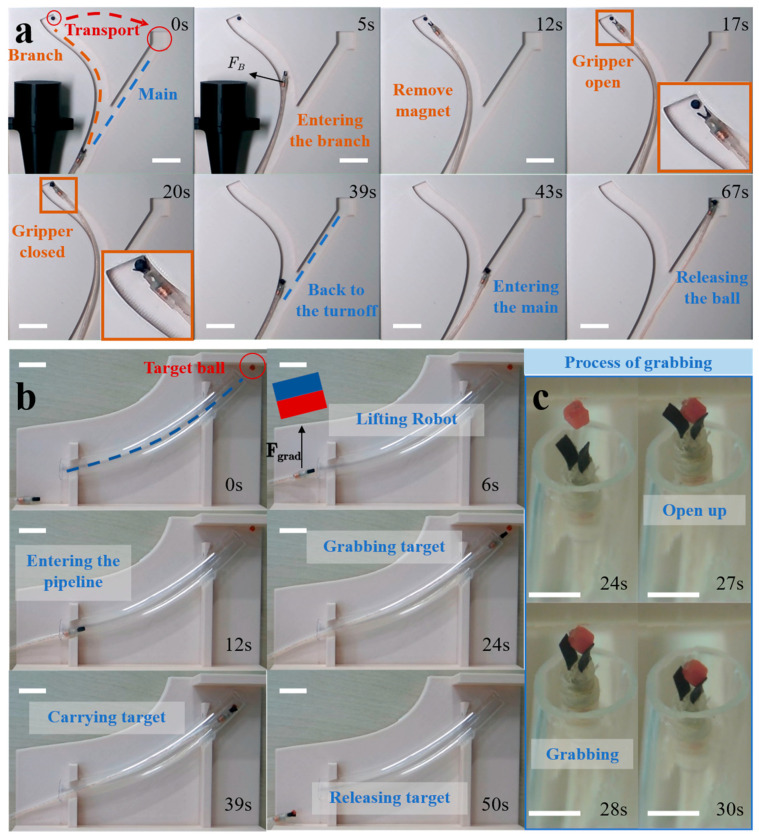
Planar and antigravity grasping experiments of the MGR: (**a**) The planar grasping experiment, where the MGR, guided by an external magnetic field, enters a branch to grasp an object and returns to the main path to release the object. (**b**) The MGR, guided by an external magnetic field, overcoming gravity to grasp and transport an object. (**c**) The detailed process of the MGR’s antigravity grasping. Scale bars, 10 mm.

To comprehensively evaluate the robot’s motion control and object-grasping capabilities in constrained environments, we constructed two sets of experimental setups designed for grasping specific targets. The first setup consists of a 3D-printed curved track plane model with a width of 10 mm, simulating the complex and restricted environments encountered in various work scenarios. As shown in Figure 6a and Movie S3, the curved track forms a “Y” shape, a common branching model. A small ball with a diameter of 3 mm is placed at the left end of the “Y” as the target object, while the right end serves as the release area.

We initially positioned a permanent magnet on the left side of the track. The magnet generates a gradient magnetic field that attracts the small ball toward the left, causing the MGR’s end to deflect leftward and enter the left track at the junction (5 s). We then pushed the MGR until it was near the ball (12 s) and applied a current to open the magnetic microgripper at the MGR’s end (17 s). Continuing to push the MGR until the gripper slightly touched the ball, we reversed the current to close the gripper and grasp the ball (20 s). Subsequently, we pulled the MGR back to the junction and pushed it forward again. Due to the right track being a straight path, the MGR will naturally enter the right track without external guidance (43 s). Upon reaching the target area, applying a current will open the magnetic microgripper, releasing the ball.

However, most work environments are not entirely flat, horizontal curved tracks. Therefore, to verify whether the MGR can perform grasping tasks effectively in three-dimensional work scenarios, we constructed a three-dimensional confined spatial experimental platform simulating a human vascular environment using a PVC pipe with an inner diameter of 10 mm. As shown in Figure 6b and Movie S4, the entrance of the pipe is slightly higher than the plane where the MGR is situated, and a 2 mm sided cube is attached above the pipe’s exit. The MGR needs to perform the grasping task against gravity.

To facilitate the MGR’s entry into the pipe, we placed a permanent magnet above the MGR, attracting it to lift to the same height as the pipe entrance (6 s). We then pushed the MGR into the pipe entrance (12 s) and removed the permanent magnet. The MGR was then guided through the pipe to reach the pipe exit (24 s). Upon reaching the working area, as shown in Figure 6c, a current was applied to open the magnetic microgripper and enclose the cube (27 s). Subsequently, a reverse current was applied to close the microgripper and grasp the cube against gravity (28 s). The MGR was then retracted until it returned to the initial platform under gravity (50 s), where the cube was released, completing the grasping task.

The above two experiments demonstrate that the MGR (magnetic gripper robot) has the capability to perform target grasping in complex bodily cavities. In further applications within the human body, it can accurately grasp tissue samples, organs, or foreign objects during tumor or abnormal tissue localization and sampling, assisting in surgical procedures. This enhances the precision, safety, and effectiveness of medical surgeries, reducing trauma and complications.

## 4. Conclusions

In this paper, we present a microgripper robot driven by distal electropermanent magnet collaboration. The MGR’s end integrates a driving system that controls the magnetic field direction via microcoils and amplifies the magnetic field strength with a permanent magnet. The microcoils enable flexible switching of the magnetic field direction, while the permanent magnet can generate a magnetic field much stronger than that of the external field or the microcoils themselves. This increases the opening and closing range of the magnetic gripper at the end, broadening its application scenarios and allowing the gripper to operate independently of external magnetic fields. Additionally, the presence of a permanent magnetic ball at the end enables the MGR to deflect towards the gradient of an external magnetic field, thereby guiding the MGR to perform minimally invasive operations within the human body.

To enable the MGR for clinical applications, we conducted a safety temperature assessment of the microcoils to ensure that their heat generation would not cause damage to the human body, allowing for safe operation. To maximize the magnetic microgripper’s grasping capability within confined spaces, we optimized the MGR’s dimensions through experimentation, determining the optimal diameter of the permanent magnetic ball and the thickness of the magnetic microgripper. We demonstrated that the magnetic microgripper can grasp objects up to ten times its own weight. Preliminary validation of the MGR’s navigation and deflection abilities in confined environments was achieved through ring experiments, while circuit experiments verified the remote control capabilities and extensibility of the magnetic microgripper. Finally, comprehensive validation of the MGR’s external field-driven guidance and end-effector grasping capabilities was conducted through planar and spatial antigravity grasping experiments. These experiments demonstrated the MGR’s ability to perform target acquisition and transportation within a complex and constrained vascular model. In the future, the MGR could be applied to sample retrieval or surgical assistance in complex in vivo environments, providing new possibilities for precision medicine.

However, before the MGR can be truly applied in clinical settings, several issues need to be addressed. For instance, during navigation, changes in the direction and gradient of the external magnetic field can exert magnetic torque and force on the magnetic microgripper, potentially interfering with its grasping ability. We can reduce disturbances by ensuring that the direction of the external magnetic field remains aligned with the gripping direction of the magnetic microgripper through methods such as magnetic positioning or visual recognition. Additionally, controlling the MGR with a gradient field is challenging and can easily damage human tissues. Developing new control platforms and algorithms to precisely manage the MGR’s deflection and movement will be crucial to ensuring the safety of clinical operations.

## Data Availability

All data needed to evaluate the conclusions in the paper are present in the paper and/or the Supplementary Materials.

## Supplementary Materials

The following supporting information can be downloaded at https://www.mdpi.com/article/10.3390/mi15060798/s1, Figure S1: Measurement for the degree of opening and closing of magnetic microgripper; Figure S2: Gripping and handling of all types of objects; Figure S3: Closing and opening distance and angle of magnetic microgrippers of different thicknesses driven by a 2 mm permanent magnet ball; Figure S4: Closing and opening distance and angle of magnetic microgrippers of different thicknesses driven by a 4 mm permanent magnet ball; Figure S5: Changes in surface temperature of silicone tube with different cooling times; Movie S1: Steering and navigation of MGR; Movie S2: Circuit connection; Movie S3: Curved track plane gripping; Movie S4: Grasping task against gravity.
