# Supplementary material for: Microgripper Robot with End Electropermanent Magnet Collaborative Actuation
Source: Micromachines (Basel). 2024 Jun 17;15(6):798. doi: 10.3390/mi15060798 (PMC11205932; doi:10.3390/mi15060798)
Supplement: Supplementary file 1 [file micromachines-15-00798-s001.zip › micromachines-3050573-supplementary/Supplementary File(s)/Supporting information.pdf]

micromachines MDPI

Supplementary Materials for

Micro Gripper Robot with End Electropermanent Magnet Collaborative Actuation

Yiqun Zhao *et al.*

Correspondence:
xinjianfan@suda.edu.cn
xiehui@hit.edu.cn

The PDF file includes:
Figures S1 to S5

Other Supplementary Material for this manuscript includes the following:
Movies S1 to S4

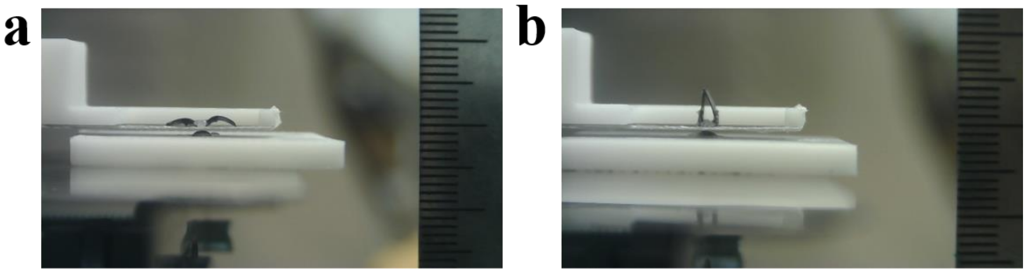

Figure s1. Measurement for the degree of opening and closing of magnetic micro gripper (**a**) Measurement of the degree of opening of the magnetic micro gripper; (**b**) Measurement of the degree of closing of the magnetic micro gripper.

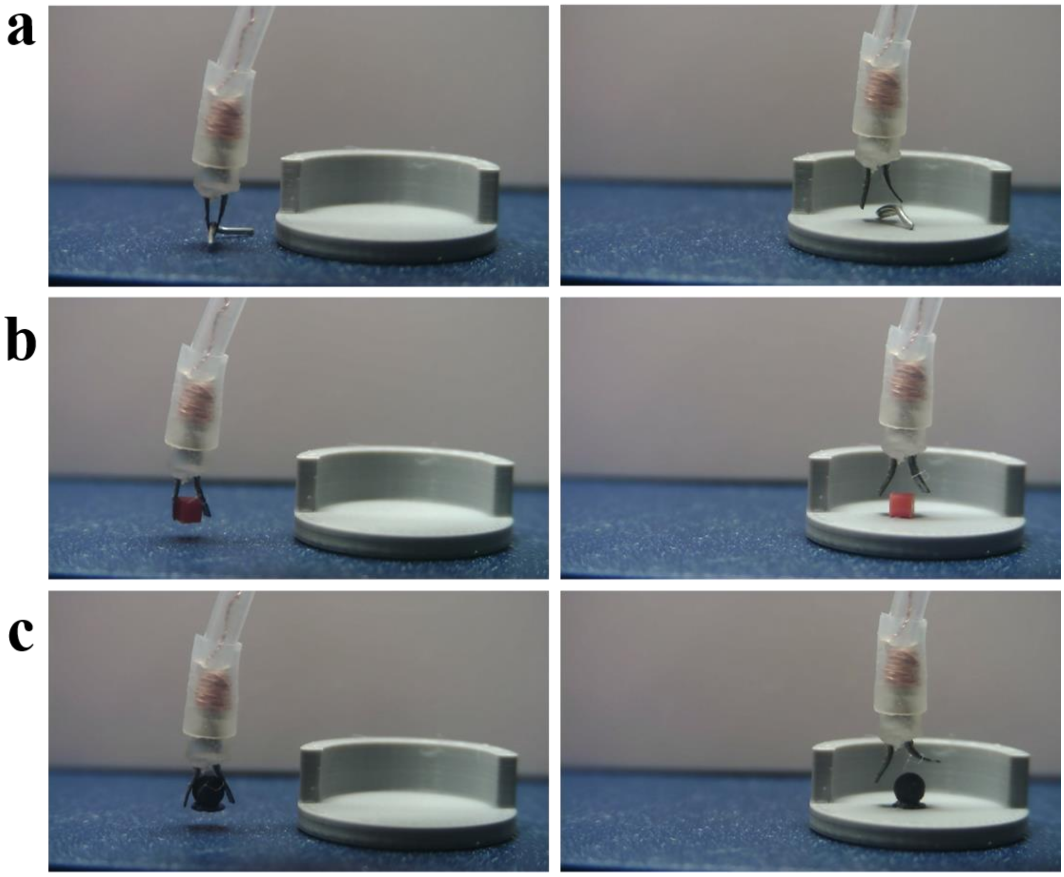

Figure s2. Gripping and handling of all types of objects. (**a**) Gripping and handling of guide wires; (**b**) Gripping and handling of squares; (**c**) Gripping and handling of spheres.

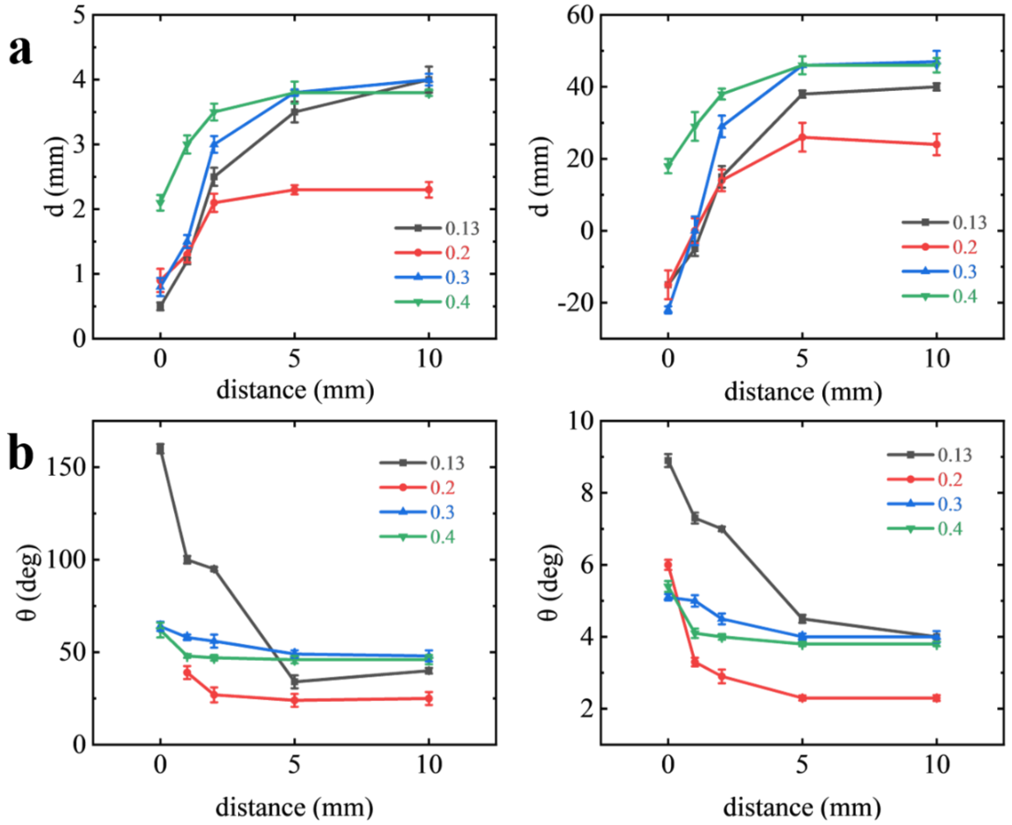

Figure s3. Closing and opening distance and angle of magnetic micro grippers of different thicknesses driven by a 2mm permanent magnet ball. (**a**) Closing and opening distance of magnetic micro grippers; (**b**) Closing and opening angle of magnetic micro grippers.

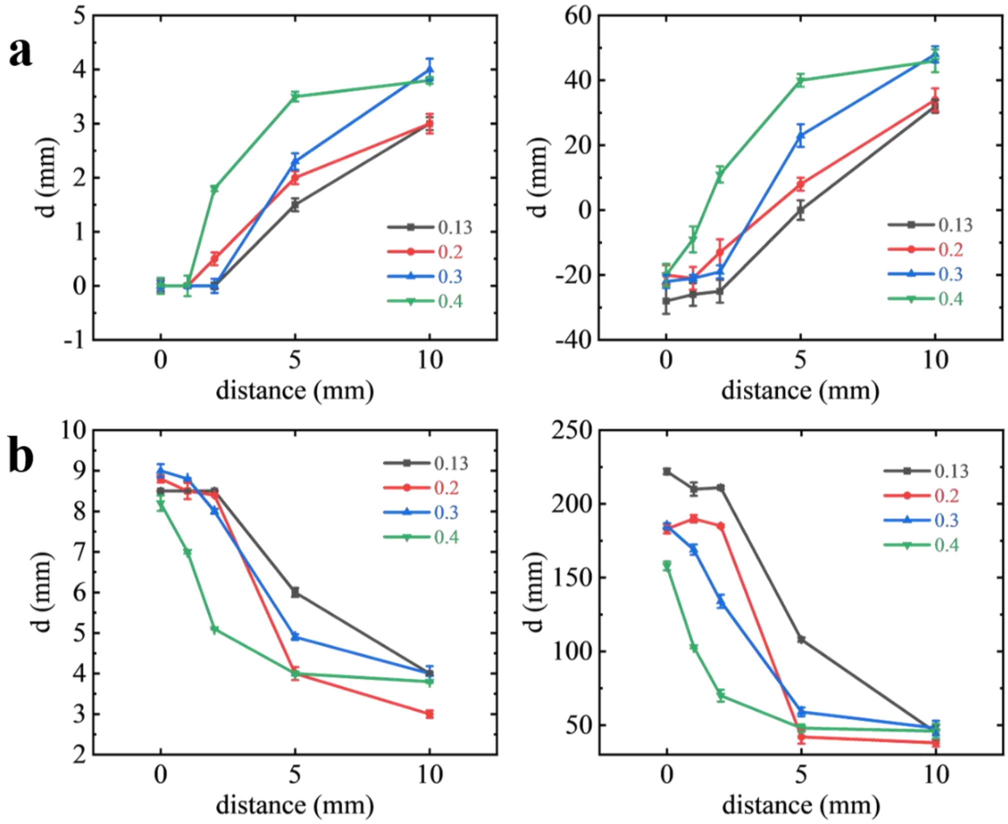

Figure s4. Closing and opening distance and angle of magnetic micro grippers of different thicknesses driven by a 4mm permanent magnet ball. (**a**) Closing and opening distance of magnetic micro grippers; (**b**) Closing and opening angle of magnetic micro grippers.

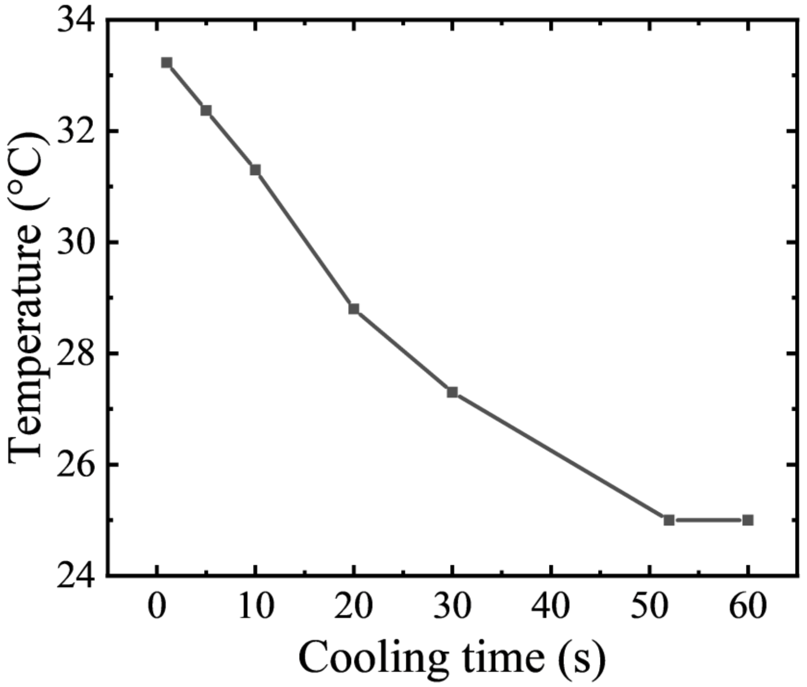

Figure s5. Changes in surface temperature of silicone tube with different cooling times
